# Protein Structure Determination in Living Cells

**DOI:** 10.3390/ijms20102442

**Published:** 2019-05-17

**Authors:** Teppei Ikeya, Peter Güntert, Yutaka Ito

**Affiliations:** 1 Department of Chemistry, Graduate School of Science, Tokyo Metropolitan University, 1-1 Minamiosawa, Hachioji, Tokyo 192-0397, Japan; 2 Institute of Biophysical Chemistry and Center for Biomolecular Magnetic Resonance, Goethe University Frankfurt, 60438 Frankfurt am Main, Germany; guentert@em.uni-frankfurt.de; 3 Laboratory of Physical Chemistry, ETH Zürich, 8093 Zürich, Switzerland; peter.guentert@phys.chem.ethz.ch

**Keywords:** protein structure determination 1, non-uniform sampling 2, spectrum reconstruction 3, structural calculation 4, paramagnetic effects

## Abstract

To date, in-cell NMR has elucidated various aspects of protein behaviour by associating structures in physiological conditions. Meanwhile, current studies of this method mostly have deduced protein states in cells exclusively based on ‘indirect’ structural information from peak patterns and chemical shift changes but not ‘direct’ data explicitly including interatomic distances and angles. To fully understand the functions and physical properties of proteins inside cells, it is indispensable to obtain explicit structural data or determine three-dimensional (3D) structures of proteins in cells. Whilst the short lifetime of cells in a sample tube, low sample concentrations, and massive background signals make it difficult to observe NMR signals from proteins inside cells, several methodological advances help to overcome the problems. Paramagnetic effects have an outstanding potential for in-cell structural analysis. The combination of a limited amount of experimental in-cell data with software for *ab initio* protein structure prediction opens an avenue to visualise 3D protein structures inside cells. Conventional nuclear Overhauser effect spectroscopy (NOESY)-based structure determination is advantageous to elucidate the conformations of side-chain atoms of proteins as well as global structures. In this article, we review current progress for the structure analysis of proteins in living systems and discuss the feasibility of its future works.

## 1. Introduction

More than 15 years have passed since Dötsch and coworkers demonstrated the first NMR spectrum of a small protein (NmerA) in living *Escherichia coli* cells in 2001 [1]. In-cell NMR has extended from prokaryotic to eukaryotic cells and has become the only tool to investigate protein behaviour inside cells at atomic resolution [2,3,4]. It has an established status as one of the biological applications of solution or solid-state NMR. So far, the method has uncovered various remarkable aspects of protein behaviour in cells or molecular crowding environments [5,6]. In intracellular or molecular crowding environments, several effects, which are generally ignored in diluted solution, such as the excluded-volume effect and nonspecific interactions, dictate protein stability and conformation. It has been proposed that the excluded-volume effect promotes compact forms of proteins [7], and that nonspecific interactions with other molecules inside cells invoke opposite effects [8,9]. Among numerous findings in the complex crowding environments, it is particularly interesting that the living cell environment notably decreases the folding stability of proteins. In-cell NMR H/D exchange experiments of human ubiquitin with three alanine mutations (L8A, I44A and V70A; referred to as ubiquitin 3A) revealed that the exchange rate of backbone amide hydrogens with solvent water was 15–20 times faster in HeLa cells than in diluted solution, demonstrating that the protein fold of ubiquitin 3A was destabilised in HeLa cells [10]. In-cell NMR studies using peculiar proteins, which were in equilibrium between a folded and unfolded conformation in diluted solution, showed that their folded states were destabilised inside mammalian and bacterial cells and the equilibrium was shifted towards the unfolded state in the cells [11,12]. Danielsson et al. [11] studied the thermodynamics of the I35A mutant of SOD1^barrel^ (superoxide dismutase 1) in mammalian (A2780) and bacterial cells, and Smith et al. [12] performed ^19^F NMR measurements for the 7-kDa globular N-terminal Src homology 3 (SH3) domain of the *Drosophila* signal transduction protein drk (downstream of receptor kinase) in *E. coli* cells. Although these are fairly different samples and experimental conditions, both suggested that destabilised proteins *in vitro* become more unstable in prokaryotic and eukaryotic cells. Moreover, intrinsically disordered proteins (IDPs) in disordered states *in vitro* persisted in disordered conditions even in the crowded cellular environment [13,14]. These results suggest that many destabilised proteins are promoted to more unstable states in intracellular environments.

While in-cell NMR is the principal tool to elucidate a protein’s natural behaviour in physiological conditions, the lack of structural information for the proteins in cells makes it difficult to fully understand the mechanisms of protein stability and details of conformational differences to those in diluted solution. In-cell NMR studies based only on chemical shift changes and peak intensities are limited to provide rough descriptions of proteins in cells based on prior knowledge. Thus, the next interest is to obtain ‘direct’ structural information for understanding how the 3D structures or dynamics of biomolecules are existing in living environments, and how they differ from the state in dilute *in vitro* solution. Despite the remarkable feature of NMR which permits to access conformational information from individual atoms of biomacromolecules and to determine their 3D conformations, 3D protein structure determinations in cells are still very few. This is due to the fact that it is still not straightforward to collect sufficient interatomic distance and angle information from in-cell NMR data because of the low signal-to-noise (S/N) ratio and an enormous number of background signals. The background signals are crucial issues particularly when using the system of intrinsic overexpression of proteins in cells [15,16], or observing ^13^C resonance spectra so that they contain many noise peaks derived from the natural abundance of ^13^C sources in cells. Despite these difficulties, several in-cell NMR studies tackled the problems and current progress allows elucidating structural details of proteins inside cells. In this article, we introduce several studies which have yielded direct structural information for biomolecules in living cells, and further determined accurate 3D structures based on measurements of 3D NOESY and triple-resonance NMR spectra. Finally, we discuss the current challenges which must be solved in the next years and feature perspectives for protein structure determination by in-cell NMR.

## 2. Paramagnetic NMR

Conventional NMR protein structure determinations usually utilise interatomic distance information derived from nuclear Overhauser effects (NOEs) [17]. However, because of abundant background signals, the short lifetime of cells in an NMR sample tube, and low concentrations of the proteins of interest, it is not trivial to record 3D NOESY-type spectra and to collect a sufficient quantity of NOE-derived distance restraints. Whilst recent progress described in the next section can overcome these problems, paramagnetic effects, such as paramagnetic relaxation enhancements (PRE), pseudocontact shifts (PCS), and residual dipolar couplings (RDC), provide alternative or complementary structural data to the NOE-derived distances [18,19,20]. They are particularly useful for unstable samples, such as living cells in an NMR sample tube, because structural information can be collected even from 2D ^1^H–^15^N or ^1^H–^13^C correlation spectra, while the NOESY-type experiments require 3D measurements to reduce overlaps among signals of the target protein or with background from cells. It should be noted that the PRE and PCS effects provide long-range structure information from a metal centre for distances up to about 40 Å [18], and RDC provides information on the orientation of bonds for scalar coupled spins relative to the static magnetic field. A drawback for observing the paramagnetic effects is the need to incorporate lanthanide-binding tags (LBTs) to the proteins unless they have a strong natural affinity for paramagnetic lanthanide ions. Either the metal-binding proteins or LBTs must have sufficiently strong affinities to lanthanide ions possessing cytotoxicity. As general issues for the observation of PCS, including *in vitro* experiments, the LBTs also require a stable, covalent linker to the protein, which should be short to limit their flexibility so as to achieve accurate structural information. The tags should maintain only one stereoisomer and minimise the structural distortions of proteins by introducing them. For in-cell NMR experiments, disulphide bonds, which are often used in chemical modifications to proteins, cannot be adopted because of the intracellular reducing environment. The same is true for the conjugating nitroxide radicals with disulphide bonds that are commonly used for obtaining PREs *in vitro*. Recently, several new LBTs have been developed for the acquisition of PRE, PCS, and RDC data inside cells [21,22,23,24,25] (Table 1). An approach to suppress the mobility of LBTs is to employ steric bulk chelators with a relatively short linker. Häussinger et al. proposed a stable LBT yielding very large PCSs (beyond 5 ppm), referred to as DOTA-M8 SPy, which is mainly composed of a DOTA (tetraxetan; 1,4,7,10-tetraazacyclododecane-1,4,7,10-tetraacetic acid) framework and eight methyl groups [26]. The eight methyl groups result in both hydrophilic and hydrophobic faces of the LBT. This allows, in addition to the covalent linker, for a secondary, noncovalent attachment to proteins due to hydrophobic interactions. The low mobility of this LBT due to the steric overcrowding and hydrophobic interactions to proteins yielded large PCSs as well as an extremely high affinity of the DOTA framework to lanthanides with a binding constant of the order of 10^−25^ to 10^−27^ M. Hikone et al. improved DOTA-M8 SPy for obtaining structural information from in-cell NMR by altering the fragile disulfide linkage of the original DOTA-M8 SPy into a carbamidemethyl (CAM) group that is stable in reducing intracellular environments (henceforth referred as DOTA-M8-CAM-I) [24]. DOTA-M8-CAM-I was attached to ubiquitin 3A with two different cysteine mutations (K6C and S57C). Incorporating the [Dy^3+^(DOTA-M8-CAM-I)]-tagged ubiquitin mutants into HeLa cells, this permitted observation of relatively large PCSs in order to obtain structural information for a protein in human cultured cells. In the meantime, Müntener et al. investigated the reactivity and stability of several LBTs possessing a DOTA-M7Py framework with a linker comprising a thioether bond that is irreversible under reducing conditions including intracellular environments [22,25]. Among them, M7PyThiazole-SO_2_Me-DOTA showed high stability, reactivity, and strong PCSs and RDCs. It efficiently reacted to more than 99% within 5 min at pH 7.0 and 295 K with a small peptide (Leu-Cys-Asp), which was the identical sequence as the tagging site of ubiquitin 3A S57C. It also tagged ubiquitin 3A S57C to an extent >95% and did not hydrolyse at pH 7.0 and room temperature. It yielded large PCSs and RDCs (up to 10 ppm and 32 Hz) with Dy^2+^ when conjugated with ubiquitin 3A S57C and K48C, and human carbonic anhydrase II (hCA-II) S166C/C206S. This high performance was attributed to the rigidity of the linker consisting of a sulphide bond between the pyridine thiazole ring and a cysteine residue that is probably due to steric clashes of the tag with the protein. Although in-cell NMR using this LBT has not been reported yet, these new stable tags for reducing environments inside cells are good candidates for obtaining accurate structural information in future in-cell NMR studies. 

While in principle PCS is one of the only few sources of reliable experimental data containing direct structural information of proteins for in-cell NMR, it is still difficult to determine *de novo* protein structures exclusively from PCS data. Comprehensive reviews of the theory and applications of PCS *in vitro* can be found in articles by Bertini, Otting, and Meiler et al. [19,30,31]. The PCS ∆*δ*^PCS^ is related to the structure and the magnetic susceptibility tensor by

(1) $$\Delta \delta^{\mathrm{PCS}}=\frac{1}{12\pi r^{3}}\left[\Delta \chi_{ax}\left(3\cos^{2}\theta -1\right)+\frac{3}{2}\Delta \chi_{rh}\sin^{2}\theta \cos2\phi \right]$$

where *r* is the distance between the lanthanide ion and the atom for which the PCS is observed, *θ* and *ϕ* are polar coordinates of the atom with respect to the magnetic susceptibility tensor ∆*χ* of the lanthanide, and ∆*χ*_ax_ and ∆*χ*_rh_ are the axial and rhombic components of ∆*χ*, respectively. It contains not only distances between a metal centre and individual atoms, but also a priori unknown magnetic susceptibility tensor parameters, as well as ambiguity due to the flexibility of the LBTs and experimental errors [32,33]. The determination of the tensor components without prior 3D structure information is not straightforward compared to the data analysis for NOEs and PREs. Also, large chemical shift differences between diamagnetic and paramagnetic data can make it difficult to achieve resonance assignments. The signals weakened by the paramagnetic relaxation enhancement effect and massive background signals hinder the analysis of these data as well. Hence, the shifted signals are generally assigned by substituting several lanthanide ions with different magnetic susceptibilities so as to gradually alter the magnitude of the shifts and depicting their trajectories in the spectra. Moreover, it is necessary to collect PCS data from several different paramagnetic centres, or mutation sites, in order to obtain sufficient structural information for most of the protein. Considering that in-cell NMR experiments require a substantial amount of protein samples and cells, it is demanding to perform sufficient PCS experiments for the resonance assignment and data collection required for a structure determination.

Thus, two distinct groups, Müntener et al. [22] and Pan et al. [23], employed an empirical approach for protein structure determination using PCS- or GPS-ROSETTA, which relies on the ROSETTA software that was originally developed for *ab initio* protein structure prediction from amino acid sequences [34,35]. At nearly the same time, the two groups performed structure determinations of *Streptococcus* protein G B1 domain (57 a.a., 7 kDa; henceforth referred to as GB1) by in-cell NMR using *Xenopus laevis* oocytes. 

Müntener et al. [22] utilized DOTA-M7Py which maintains several remarkable features: (1) strong affinity (*K*_d_ < 10^−25^ M) toward lanthanides due to the DOTA framework, (2) exclusively the square antiprismatic Λ(δδδδ) stereo-configuration for the 4S,3R-Lu derivative, (3) a short linker with a nonreducible thioether bond, and (4) both hydrophilic and hydrophobic properties for reducing mobility in a similar manner as DOTA-M8 SPy. The measurements of in-cell PCSs and RDCs using this tag were achieved by microinjection into *Xenopus* oocytes for an intracellular concentration of GB1 of about 50 µM. The PCSs of ^1^H and ^15^N, and ^1^H–^15^N RDCs were respectively collected for Tm^3+^ and Tb^3+^ at three Cys-mutation sites. Because ROSETTA has the ability to predict 3D protein structures from sequences, the authors tested the prediction performance of GPS-ROSETTA under three input conditions: no experimental data, only PCS, and PCS and RDC. Their results demonstrated that GPS-ROSETTA using the PCS and RDC data provided accurate global structures of GB1 in *Xenopus* oocytes with a C^α^ RMSD of 0.64 Å to the crystal structure (Protein Data Bank; PDB code: 2QMT), which was not possible by structure prediction with ROSETTA from the sequence alone. They concluded that this approach was sufficiently accurate to determine well-defined protein structures, and the overall structural features of GB1 in Xenopus oocytes were similar to those observed *in vitro*. 

Pan et al. [23] performed the 3D structure determination with PCS in *Xenopus* oocytes using GPS-ROSETTA, by a similar approach as described above except that the LBT 4PhSO_2_-PyMTA [27] was used. Features of the commercially available 4PhSO_2_-PyMTA are that it has a short and stable thioether bond in the linker to the protein, and a simple chemical structure compared to the compounds with the DOTA-framework. Using PCSs at two Cys-mutation sites with Tb^3+^, Tm^3+^, and Yb^3+^, they performed the modelling of the GB1 structures in cells by GPS-ROSETTA. The structure by ROSETTA with the PCS data has a C^α^ RMSD of 1.0 Å from the crystal structure (PDB code: 2QMT) and the 25 lowest energy structures were a well-converged with less than a C^α^ RMSD of 0.15 Å from the lowest energy one. The method presented sufficiently accurate structures of GB1 which remained unchanged in the cellular environment despite the notable structural variations for residues 8–12 in a loop. Hence, these results demonstrated by the two groups suggest that the combination approach of in-cell data with software for *ab initio* protein structure prediction, such as ROSEETA, would be a powerful tool to visualise 3D protein structures inside cells.

Compared to the difficulty for the collection and analysis of PCS data of in-cell NMR, the acquisition of structural information from PRE may be advantageous from some perspectives [19,36]: (1) several chemically stable tags under reducing environment are commercially available, (2) the calibration of interatomic distance from PRE data is simpler due to its isotropic effect, and (3) PREs are relatively tolerant against tag flexibility compared to PCS [19,36]. In the case of a hydrogen atom, the intensity ratio of a particular proton in paramagnetic/diamagnetic spectra, $I_{\mathrm{para}}/I_{\mathrm{dia}}$, is approximated from the transverse relaxation rates of a diamagnetic and paramagnetic spin, $R_{2}$ and $R_{2}^{\mathrm{sp}}$, respectively [37]:

(2) $$\frac{I_{\mathrm{para}}}{I_{\mathrm{dia}}}=\frac{R_{2}\;\exp\left(-R_{2}^{\mathrm{sp}}t\right)}{R_{2}+R_{2}^{\mathrm{sp}}}$$

where *t* is the total INEPT (insensitive nuclei enhanced by polarization transfer) evolution time in the case of a heteronuclear single quantum coherence (HSQC) spectrum. The paramagnetically enhanced transverse relaxation rate $R_{2}^{\mathrm{sp}}$ is converted into a distance from the metal centre by use of the following equation:

(3) $$R_{2}^{\mathrm{sp}}=\frac{K}{r^{6}}\left(4\tau_{c}+\frac{4\tau_{c}}{1+\omega_{h}^{2}\tau_{c}^{2}}\right)$$

where the constant *K* is 1.23 × 10^−32^ cm^6^ s^−2^, *r* is the distance between a lanthanide ion and a hydrogen atom, *τ_c_* is the correlation time for the lanthanide, and *ω_h_* is the Larmor frequency of the proton. Although *R*_2_ and *τ_c_* have to be estimated from additional relaxation experiments to be precise, in-cell NMR studies usually employ approximated values obtained from the line width at half-height of peaks for simplicity. This approximation makes it easy to convert PRE data into distance restraints albeit it sacrifices some of their accuracy. It is also advantageous that some tags for PRE measurements in cells are commercially available. Theillet et al. [28] attached a DOTA-maleimide tag with a Gd^3+^ ion to a single cysteine of a representative IDP, α-synuclein, and observed PREs in human cultured cells [28]. The incorporation of LBTs into proteins via maleimide coupling is chemically stable in reducing environments. In the article, the authors showed that the α-synuclein conformation in cells is similarly compact as *in vitro* and that the character of intrinsic disorder is sustainable inside cells. 

## 3. *De Novo* in-Cell Protein Structure Determination

### 3.1. De novo Structure Determination in Prokaryotic Cells

As described above, in-cell NMR has made it possible to obtain structural information in order to infer partial conformations of proteins in cells with prior knowledge. Meanwhile, it is also indispensable for the analysis of protein behaviour in cells to accomplish complete structure determinations including side-chain atoms. For instance, side-chain conformations are thought to be predominantly affected by the intracellular environment, which allows to understand the functions of proteins and extend the method to other applications such as structure-based drug discovery. It remains necessary to achieve *de novo* 3D protein structure determination in living cells using NOE-derived distance restraints between side-chains. However, as already mentioned, there are several obstacles to collect a sufficient number of distance restraints from NOESY-type spectra in intracellular environments, e.g., the low concentration of target molecules in cells, the short lifetime of cells, severe background signals from other components of the cells, etc. To date, NMR observations of isotopically labelled-proteins inside cells have been achieved principally by two approaches: intrinsic overexpression of proteins in cells [1,16,38,39] and incorporation of stable isotope-labelled molecules by importing them through the cellular membrane [10,28,40]. The system of intrinsic protein overexpression has advantages in terms of the protein concentration in cells and practical experimental simplicity. For instance, the concentration of the *T. thermophilus* HB8 TTHA1718 gene product (66 a.a., 7 kDa) could reach up to 3–4 mM in *E. coli* cells [15]. It also allows regulation of the protein concentration by altering the delay time after induction and the incubation temperature to some extent [41]. In addition, the method can easily repeat in-cell NMR measurements owing to the omission of protein purification steps. Although the background signals derived from cells are a critical issue in this approach, the desirable features permitted to measure ^13^C-separated, ^15^N-separated NOESYs, and 3D triple-resonance spectra for backbone and side-chain resonance assignments. Indeed, protein structure determinations by in-cell NMR currently reported have been achieved exclusively by the system of intrinsic protein overexpression. The first *de novo* protein structure determination by in-cell NMR utilised an overexpression system in *E. coli* [41]. The short lifetime of cells in an NMR sample tube was a challenge for the measurement of NOESY spectra, because *E. coli* cells died or started to release target proteins within approximately 6 h, while it generally takes a couple of days to record 3D NMR spectra with high S/N ratio and resolution. Among the many approaches to address this issue, the most robust and straightforward method is to utilise nonuniform sampling (NUS) in combination with spectral reconstruction by non-Fourier transform methods [42]. The sparse sampling by NUS allows to cover the entire original experimental data matrix ensuring sufficient peak intensity within the cell lifetime. In the first in-cell structure determination, the NUS data was reconstructed by interpolating the missing points with a maximum entropy (MaxEnt) approach [43,44]. This enabled the assignment of the backbone and a majority of the side-chain atoms, as well as collecting a sufficient number of NOE distance restraints for the protein TTHA1718. The resulting structure was well-defined with a backbone root-mean-square deviation (RMSD) below 1.0 Å and is similar to the *in vitro* structure with a backbone RMSD between the two structures of 1.2 Å. Slight structural differences were observed in the putative heavy metal binding loop where chemical shift differences between in-cell and *in vitro* reflected possible metal binding. The authors discussed that the interactions with metal ions in the *E. coli* cytosol or the effects of viscosity and intracellular molecular crowding might affect the conformation of this region. 

**Figure 1:**
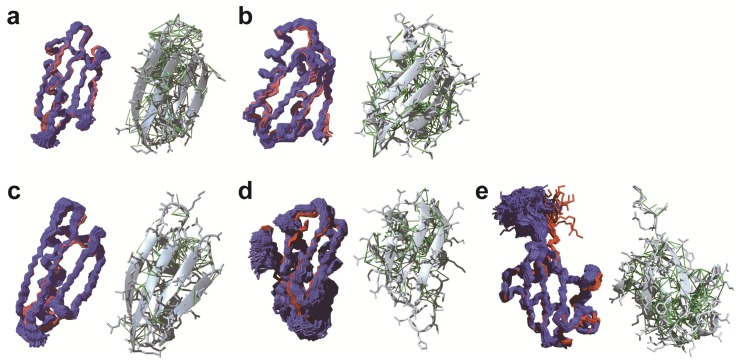
Protein structures determined by in-cell NMR. GB1 (**a**; PDB code: 2N9L) and TTHA1718 (**b**) structures in *E. coli* cells. GB1 (**c;** 5Z4B), TTHA1718 (**d**; 6K1V), and ubiquitin 3A mutant (**e**; 6K1U) structures in sf9 cells. For all structures, the left panels show the backbones of the structure ensemble in cells (blue) and in diluted solution (red). The right panels show the best conformer in cells with side-chains (grey) and NOE distance restraints (green).

Later on, the same group improved the procedure for the *de novo* in-cell protein structure determination with three methodological advances composed of improved NMR data processing of NUS data, automated chemical shift assignment, and robust structure calculation with Bayesian inference [45]. The new procedure permitted 3D protein structure determinations with much lower intracellular protein concentrations and even without indirect restraints such as hydrogen bond information. The structure of the protein GB1 in living *E. coli* cells was determined at an order of magnitude lower concentration (approximately 250 μM) in the NMR tubes than in the original report for TTHA1718 (3–4 mM). This is comparable to a physiologically natural environment, where the maximal natural concentration of a protein in normal cells is a few dozen to hundreds of μM [46,47]. The NMR data processing for the indirect dimensions of 3D NMR spectra employed the Quantitative Maximum Entropy (QME) method [16] instead of the conventional 2D MaxEnt implemented in the program Azara [48] that had been used for the previous structure determination. Chemical shifts of in-cell GB1 were assigned by combining conventional manual analysis with an automated assignment procedure using the FLYA (fully automated assignment) algorithm [49]. A remarkable feature of FLYA is that it enables resonance assignments exclusively from NOESY-type NMR spectra [50,51,52]. Using NOESY spectra was crucial for obtaining side-chain assignments because faster transverse relaxation of in-cell samples hinders the collection of a sufficient number of signals from through-bond spectra, e.g., H(CCCO)NH, for side-chain resonance assignments. NOESY spectra, on the other hand, contained a considerable number of signals from the side-chains. Structure calculations were performed employing the program CYANA (combined assignment and dynamics algorithm for NMR applications) with the CYBAY (CYANA Bayesian inference) module [53], which was able to extract a maximum of structural information from the limited and ambiguous experimental NOESY data collected in living cells. The GB1 structure ensemble of 1900 conformers calculated by CYBAY is well defined with an average backbone RMSD of 0.43 Å to the mean coordinates (Figure 1). The backbone RMSD between its mean structure and the *in vitro* structure was 1.18 Å. A loop and the end of a β-strand (residues 11–14) showed low RMSDs to the *in vitro* structure for the C^α^ atoms but higher RMSDs of more than 2.0 Å for the side-chains. These residues coincided well with a region of slightly higher chemical shift differences between the in-cell and *in vitro* samples (residues 10–13). The authors discussed that the structural changes of the side-chains might be due to molecular crowding effects or the intracellular environment, in which the interactions with other negatively charged molecules might result in the structural changes of side-chains. The improved method yielded in-cell TTHA1718 structures that were much better defined than before owing to the additional distance restraints identified by the FLYA analysis of the quantitative maximum entropy (QME)-processed NOESY spectra and the improved distance accuracy by Bayesian inference (Figure 1). The study also confirmed that structural differences were located in three dynamics loop regions (residue 9–12, 26–29 and 44–50) of TTHA1718, which may be affected by the viscosity and macromolecular crowding in the cytosol.

### 3.2. De novo Structure Determination in Eukaryotic Cells

Until very recently, in-cell *de novo* structure determinations were performed exclusively in *E. coli* cells, presumably because the achievable target protein concentration in eukaryotic cells was too low to obtain a sufficient number of NOE-derived distance restraints. In 2019, Tanaka et al. showed the first *de novo* protein structure determinations in living eukaryotic cells using a sf9 cell/baculovirus system, which are based exclusively on information from 3D heteronuclear multidimensional NMR spectra [54]. As model systems, three small- and two medium-sized proteins were chosen: GB1, TTHA1718, ubiquitin 3A, rat calmodulin (148 a.a., 17 kDa), and C-terminally truncated human HRas (residues 1–171, 19 kDa). The concentration of GB1 protein in the sf9 samples was estimated as 50 ± 12 μM, which is nearly the maximum protein concentration in intracellular environments, but too low to determine 3D structures by the previous methods. Thus, the authors applied the bioreactor system supplying fresh medium into the NMR tube continuously during the measurements [55,56,57] to prolong the lifetime of the cells, and hence the NMR measurement time, to at least 24 h with over 90% cell viability. The bioreactor system also removed extracellular proteins released from the cells, ensuring that the in-cell NMR spectra were obtained only from protein inside sf9 cells. All 3D NMR data were sparsely measured by a sampling scheme with a sinusoidal-weighted Poisson distribution, so-called Poisson-gap sampling [58], in order to minimally suppress missing information from large gaps between data points and biased distributions. Subsequently, data were reconstructed by QME. The measurement and processing scheme with the bioreactor, sparse sampling, and QME reconstruction improved significantly the sensitivity of the in-cell spectra. The quality of these spectra allowed to collect NOE-derived distance restraints sufficient for determining high-resolution 3D structures. 

For GB1 in sf9 cells, 3D triple-resonance NMR spectra could be measured with high quality for backbone and side-chain resonance assignments, and unambiguous assignments were achieved for approximately 98% of the backbone resonances of GB1 in sf9 cells. The Bayesian inference-assisted structure refinement well defined ensemble structures of GB1 with an average backbone RMSD of 0.51 Å to the mean coordinates (Figure 1). The backbone RMSD between the mean structures in sf9 cells and in diluted solution was 1.61 Å, except for a region composed of a loop and an α-helix (residues 22–26, 28) that shows higher RMSD values around 1.5 Å. These residues coincided well with a region exhibiting chemical shift differences between the in-cell and diluted solution samples (residues 20–24, 27). The relative position of the α-helix in-sf9 structures was tilted away from the β-sheet. The authors concluded that the changes in chemical shift and 3D structure for this region, which interacts through a hydrophobic patch on the protein surface with other molecules nonspecifically, were presumably due to the effects caused by the intracellular environment. A structural difference compared to the structure in diluted solution was also observed in a similar region of the GB1 structure in *E. coli* cells (residues 20–24) [45], and a molecular dynamics study simulating crowded environments [59] suggested that the intracellular environment perturbed the conformation of the region similarly in *E. coli* and sf9 cells. It is interesting to note that the method achieved the observation of minor structural differences even from in-cell NMR spectra with low S/N ratio. However, GB1 is currently the only protein whose structure has been very accurately determined in living eukaryotic cells. Verifications of this finding for other proteins may be needed. In order to elucidate structural differences more quantitatively, it is also essential to validate the in-cell structures in various ways, such as by measuring paramagnetic effects and observing structural dynamics in cells with NMR relaxation experiments.

Structure determinations of Ub3A and TTHA1718 were also performed with distance restraints obtained from 3D NOESY spectra in sf9 cells. The chemical shift assignments for these proteins were transferred from the data in diluted solution based on the knowledge that chemical shift differences for these proteins were small between sf9 cells and diluted solution. The resulting structure ensemble of Ub3A was well-defined with an average backbone RMSD of 0.39 Å to the mean coordinates, and 1.31 Å to that in diluted solution (Figure 1). The structure ensemble of TTHA1718 presented an average backbone RMSD of 0.88 and 2.60 Å to the mean coordinates and to that in diluted solution, respectively. The relatively large RMSDs of TTHA1718 were attributed to the putative metal-binding loop region of residues 9–18, for which only few NOE distance restraints were collected, presumably due to exchange processes related to the binding of various metal ions. Excluding this region, the backbone RMSD to the structure in diluted solution was 1.27 Å.

For calmodulin and HRas in sf9, samples were prepared with methyl- and aromatic-selective ^1^H/^13^C- labelling to reduce signal overlap. In both cases, well-resolved 3D ^13^C-separated NOESY spectra were acquired, indicating that this method is effective for obtaining NOE-derived structural information of proteins with molecular weight over 15 kDa in eukaryotic cells. Comparison of in-cell NMR spectra of calmodulin with those in diluted solution suggested that calmodulin in sf9 cells maintains a conformational state similar to the Mg^2+^-bound form in diluted solution. This data indicates that the bioreactor system successfully kept sf9 cells healthy and suppressed the stress-induced increase of the intracellular Ca^2+^ concentration, which has been reported in a previous in-cell NMR study using HeLa cells [60]. Spectra of HRas expressed in sf9 cells were similar to those in the ‘inactive’ guanosine diphosphate (GDP)-bound state, suggesting that the C-terminally truncated human HRas cannot be activated to the guanosine triphosphate (GTP)-bound form at the cell membrane, while GTP molecules bound to HRas during protein synthesis in sf9 will be hydrolysed by its intrinsic GTPase activity during the incubation period. Finally, the authors concluded that backbone and side-chain resonance assignments and 3D structures of proteins of less than 10 kDa size can be determined exclusively from NOE-derived distance restraints acquired in living sf9 cells. For medium-sized proteins, such as calmodulin and HRas, it is possible to obtain high-resolution structural information from in-cell NOESY experiments in combination with selective ^1^H/^13^C-labelling. 

## 4. Conclusions and Outlook

In this article, we focused on in-cell NMR studies that yielded direct structural information or determined protein 3D structures in living systems. The experimental and computational techniques outlined in this article provide insight into the 3D structural information for a variety of biological functions in living systems. The paramagnetic effects, such as PRE, PCS, and RDC, provide highly promising data, while NOE-based structure determination is crucial to delineate detailed protein conformations including side-chain atoms. Despite the high interest in studying protein conformations with atomic resolution in cells, to the best of our knowledge, these studies are still very few and limited to a small number of proteins. It might imply that these are delicate methods that are too demanding and laborious for routine applications to many proteins and associated functional studies. However, incorporating the current progress described here, we expect 3D structure analysis by in-cell NMR to play a significant future role in elucidating functionally relevant structure and dynamics at atomic resolution in living cells. Moreover, stereospecific isotope labelling [61,62] and segmental labelling techniques [63,64,65,66,67] would be valuable for future applications of in-cell NMR. State-of-the-art computational methods in automatic resonance assignment using a limited number of spectra [51,52], protein 3D structure prediction [68], sparse modelling such as compressive sensing [69,70,71,72], machine learning [73], and molecular dynamics simulation [74] will also contribute to the advancement in this field. Over time, these methods will jointly help to elucidate the functions and behaviour of biomolecules in living systems and find potential application in structure-based drug screening and clinical therapy. 

## Figures and Tables

**Table 1:** Lanthanide-binding tags (LBTs) proposed for inducing paramagnetic effects in living cells.

Name	Chemical Structure ^1^	Reported Paramagnetic Effects	Linker	Reference	Commercially Available [CAS] ^2^
DOTA-M8-CAM-I	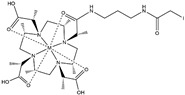	PCS	*N*-propylene-acetamide	[24]	no
4PhSO_2_-PyMTA	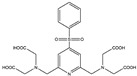	PCS/PRE	pyridine	[23,27]	yes
DOTA-maleimide	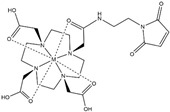	PRE	*N*-ethylene-maleimide	[28]	yes [1006711-09-5]
DOTA-M7Py	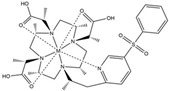	PCS	pyridine	[22]	no
DO2A	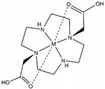	solvent PRE	―	[29]	yes [112193-75-6]
M7PyThiazole-SO2Me-DOTA	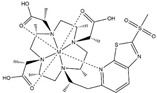	PCS/RDC	pyridine thiazole	[25]	no

^1^ M denotes lanthanoid ions, ^2^ Chemical Abstracts Service (CAS) registry number, if available.

## Abbreviations

HSQC	Heteronuclear Single Quantum Coherence
H/D	Hydrogen/Deuterium
NUS	Non-uniform Sampling
SOD	Superoxide Dismutase
PCS	Pseudo-Contact Shift
RDC	Residual Dipolar Coupling
IDP	Intrinsically Disordered Protein
LBT	Lanthanide-Binding Tag
NUS	Non-Uniform Sampling
NOE	Nuclear Overhauser Effect
GB1	protein G B1 domain
PRE	Paramagnetic Relaxation Enhancement
PDB	Protein Data Bank
